# Identification and Characterization of a Rhodopsin Kinase Gene in the Suckers of *Octopus vulgaris*: Looking around Using Arms?

**DOI:** 10.3390/biology10090936

**Published:** 2021-09-19

**Authors:** Al-Sayed Al-Soudy, Valeria Maselli, Stefania Galdiero, Michael J. Kuba, Gianluca Polese, Anna Di Cosmo

**Affiliations:** 1 Department of Biology, University of Naples Federico II, Via Cinthia 26, 80126 Naples, Italy; alsayedalsoudymohamed.mostafa@unina.it (A.-S.A.-S.); valeria.maselli@unina.it (V.M.); gianluca.polese@unina.it (G.P.); 2 Department of Pharmacy, School of Medicine, University of Naples Federico II, Via Domenico Montesano 49, 80131 Naples, Italy; stefania.galdiero@unina.it; 3 Department of Neurobiology, Hebrew University of Jerusalem, P.O. Box 12271, Jerusalem 91120, Israel; michi.kuba@me.com; 4 Physics and Biology Unit, Okinawa Institute of Science and Technology Graduate University (OIST), 1919-1 Tancha, Onna-son, Okinawa 904-0945, Japan

**Keywords:** cephalopods, octopus, arm suckers, skin, retina, extra-ocular perception, GRK1

## Abstract

**Simple Summary:**

Octopus arms are a fascinating and evolutionarily unique sensory organ, with hundreds of motile suckers, each with thousands of sensory cells, lining eight highly flexible arms. Scientifically, there are many open questions regarding the sensory capabilities of the arms and specifically the highly innervated suckers. In our present work, we used a multidisciplinary approach to fully characterize the light-sensing molecule, Ov-GRK1, in the suckers, skin and retina of *Octopus vulgaris*. We sequenced the *O. vulgaris* GRK1 gene, defining a phylogenetic tree and performing a 3D structure model prediction. We found differences in the relative expression of mRNA in different sucker types at several locations along the arm, which might indicate a functional difference. Using labeling methods, we localized the expression to the highly sensitive sucker rim. Our findings indicate that octopus suckers, in specific areas of the arm, might have the ability for light sensing. We therefore suggest that suckers are tactile, chemical and light sensors.

**Abstract:**

In their foraging behavior octopuses rely on arm search movements outside the visual field of the eyes. In these movements the environment is explored primarily by the suckers that line the entire length of the octopus arm. In this study, for the first time, we report the complete characterization of a light-sensing molecule, Ov-GRK1, in the suckers, skin and retina of *Octopus vulgaris*. We sequenced the *O. vulgaris* GRK1 gene, defining a phylogenetic tree and performing a 3D structure model prediction. Furthermore, we found differences in relative mRNA expression in different sucker types at several arm levels, and localized it through in situ hybridization. Our findings suggest that the suckers in octopus arms are much more multimodal than was previously shown, adding the potential for light sensing to the already known mechanical and chemical sensing abilities.

## 1. Introduction

Cephalopods are known for their rapid camouflage ability; they adapt their appearance based on visual observation of the surrounding environment and potential predators or prey. Though many aspects of this behavior are still unknown, generally, visual input from the eyes is processed in their optic lobes and central brain, from which motor output signals are sent to chromatophores, iridophores, leucophores, and papillae, resulting in color, pattern and/or shape change [1,2,3,4]. Even before hatching, once rhabdomeres are present in the retina, embryos exhibit a behavioral response to light stimulation [5]. In addition to ocular visual sensing, cephalopods also possess extra-ocular photoreceptors [6,7,8,9,10,11,12,13,14,15]. Mäthger et al. [13] identified the presence of rhodopsin transcripts in fin and mantle tissue of the cuttlefish, *Sepia officinalis*, suggesting that cephalopods may have dermal photoreceptors that function using the same phototransduction pathway as those in the retina. In *Octopus bimaculoides,* researchers found expression of r-opsin in the skin, and described light-activated chromatophore expansion [16]. Kingston and colleagues [14,17] identified the presence of mRNA transcripts and proteins (rhodopsin, retinochrome, and Gqa) involved in phototransduction in cephalopods’ chromatophore organs and fin muscles. Surprisingly, in hatchings *Dorotheutis pealei* small hair cells, thus far known as mechanoreceptors, co-expression of rhodopsin and retinochrome suggested a multimodal sensory function, working simultaneously as mechanical and light sensitive structures [17]. The same authors found rhodopsin and retinochrome in the arm ganglia and the sucker peduncle nerves too, suggesting that also these tissues work in a multimodal way detecting tactile and photic information. Very recently, it has been shown that the octopus arm can react to a light stimulus moving it in a reflex-like phototactic response without ocular visual input [18].

In the octopus, the arm suckers have unique features to perform a remarkable variety of tasks [19], such as anchoring the body to the substrate and grasping and manipulating objects [20,21]. They also contain extremely effective chemical and tactile sensory systems [22,23,24]. Most of the sensory cells associated with these functions are morphological modifications of the ciliated bipolar cells, which represent a common neuroreceptor archetype throughout the animal kingdom [24]. In the octopus sucker rim, 4 types of primary receptor cells were described [22]. Based on their morphology, Graziadei and Gagne [24] hypothesized their role in chemical, tactile and photoreception. Recently, van Giesen and colleagues [25] described the molecular basis of the chemo tactile system in the arm suckers. The molecular characterization and function of photoreceptor types in the arm suckers remained unclear.

The opsin family is a multigenic family of G protein-coupled receptors (GPCR) [26,27,28] and in eumetazoans there are at least 9 opsins paralogs [28], 6 of which have been identified in mollusks, and of these only 4 have been detected in cephalopods, in the genome of *O. bimaculoides*: rhodopsin, rhabdomeric opsin, peropsin, and retinochrome [29].

Rodopsin’s role in vision is well known; upon absorption of light, it activates a G-protein cascade that generates an electrical response at the surface membrane of the retinal rod cells. This response encodes the absorption of single photons, and upon transfer through the visual pathway it ultimately elicits visual sensations [30]. It consists of an apoprotein opsin and 11-cis-retinal chromophore bound by a Schiff-base linkage [31,32].

In the current study, we sequenced the *O. vulgaris* Rhodopsin_kinase gene (GRK1), defining a phylogenetic tree and performing a 3D structure model prediction. We show, for the first time, that the *O. vulgaris* GRK1 gene is expressed in the sucker rim epithelium, in addition to its expression in the retina and skin. Furthermore, by quantifying the relative mRNA in different sucker types at several arm levels, we show that expression is not uniform throughout the octopus arm. Taken together, our data suggest a light-sensing ability to octopus suckers, adding to their known functions in touch/chemo sensation [25,33]. As different areas of the arm are often used differently, differences in relative expression throughout the arm might also indicate a functional importance of suckers from certain arm areas as extra-ocular photosensors.

## 2. Materials and Methods

### 2.1. Ethical Statement

Adult specimens of *O. vulgaris* (body weight 800 g ± 50 g, mean ± SD) were collected from the Bay of Naples (Italy) and transferred to the Di Cosmo’s cephalopod facility at the Department of Biology, University of Naples Federico II (Italy). Our research was approved following the European Directive 2010/63 EU L276, the Italian DL. 4/03/2014, no. 26 and the ethical principles of Reduction, Refinement, and Replacement (Project n° 608/2016-PR-17/06/2016; protocol n°DGSAF 0022292-P-03/10/2017).

### 2.2. Tissue Collection and Fixation

**Figure 1:**
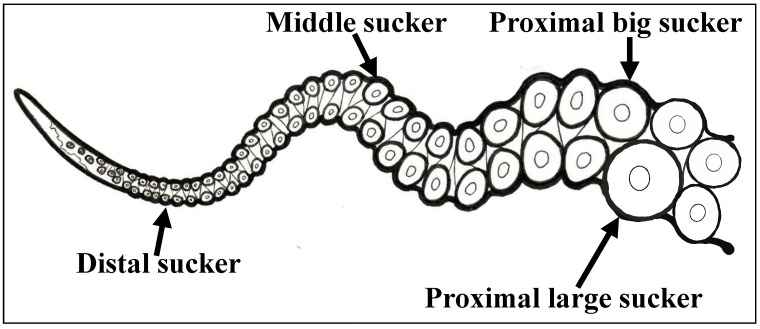
Image description of four sucker types: proximal big, proximal large, middle, distal.

Adult specimens of *O. vulgaris (n = *3, males) were anesthetized by isoflurane insufflation [34], and tissues were dissected under sterile conditions following institutional guidelines. For RNA gene analysis, we extracted tissue from four sucker types that differ in size and location on the arm (proximal big, proximal large, middle, distal; Figure 1), skin from the dorsal side of arms, retina, and heart (as negative control). The collected samples (3 samples for each tissue type) were snap-frozen and put in Trizol, then stored at −80 °C for further experiments.

To localize the expression of Ov GRK1 we performed whole mount in situ hybridization on suckers from the L1 arm. The dissected suckers were fixed overnight in 4% PFA (4% Para-formaldehyde in PBS, pH 7.4) at 4 °C. Fixed tissues were dehydrated in a graded methanol series (25% MeOH; 50% MeOH; 75% MeOH and 100% MeOH) with 1X PBST (phosphate-buffered saline with 0.1% Tween-20) for 15 min each, then stored in 100% methanol at −20 °C until use. 

### 2.3. Expression Analysis of Rhodopsin Kinase in Different Tissues and Sequencing

The analysis of the sequencing confirmed the identity of the fragments. All sequence data generated in this study were deposited in GenBank (accession numbers MW483824).

Total RNA was extracted using the RNeasy minikit (Qiagen, GmbH Valencia, CA, USA), following the manufacturer’s protocol. The quality and amount of purified RNA were analyzed spectrophotometrically with Qubit 3.0 (Thermo Scientific Inc., Waltham, MA, USA). RNA of 1000 ng was reverse transcribed with the QuantiTect^®^ Reverse Transcription Kit (Qiagen, GmbH Valencia, CA, USA). Specific PCR primers were designed with the software Geneious 9.1 (Biomatters, Auckland, New Zealand, available from http://www.geneious.com, accessed on 30 January 2021), using the coding sequence for the Rhodopsin kinase (GRK1) gene from the genome of *O. bimaculoides* [29] (Table 1).

PCRs were performed in a final volume of 20 μL, with 0.2 μL of Pfu DNA polymerase (Thermo Scientific), 4 μL of 4× Tris buffer with MgCl_2_, 1.6 μL of dNTPs (each dNTP 2.5 μM), 0.2 μL of 50 μM of each primer, and 100 ng of cDNA template under the following conditions: an initial denaturing step at 98 °C for 3 min; 35 cycles of 10 s at 98 °C; 30 s at 60 °C and 1 min at 72 °C; and a final extension step of 5 min at 72 °C. PCR products were purified from unincorporated primers using Exonuclease I and Fast Alkaline Phosphatase (Thermo Scientific). The sequencing reaction was performed using the BigDyeTM Terminator Cycle Sequencing chemistry (Applied Biosystems, Foster City, CA, USA). Sequences were purified using DyeEx 2.0 Spin Kit (Qiagen, GmbH Valencia, CA, USA) and analyzed by an ABI 3100 automated sequencing instrument (Perkin-Elmer, Genetic Analyzer, Foster City, CA, USA). Chromatograms were assembled and analyzed using software Geneious version 9.1. PCR products were analyzed with GenBank BLASTn and BLASTx (BLAST, basic local alignment search tool). Additionally, we performed a real-time PCR on four sucker types (proximal big, proximal large, middle, and distal), skin, retina from the eye, and heart (as negative control), using the QuantiTect SYBR Green PCR Kit (Qiagen, GmbH Valencia, CA, USA). PCR was performed in a final volume of 25μL, with 50 ng of cDNA, 1 mM of each primer, and 12.5μL of QuantiFast SYBR Green PCR Master Mix (2×). The PCR cycling profile consisted of a cycle at 95 °C for 5 min, 40 three-step cycles at 95 °C for 15 s, at 60 °C for 20 s, and at 72 °C for 20 s. Quantitative RT-PCR analysis was conducted by using the 2^−(∆∆Ct)^ method [35]. RT-PCR was performed in a Rotor-Gene Q cycler (Qiagen, GmbH Valencia, CA, USA). The ubiquitin gene was used for the normalization of the relative expression (Table 1). At the end of each test, a melting curve analysis was done (plate read every 0.5 °C from 55 to 95 °C) to determine the formation of the specific products. Each sample was tested and run in duplicate.

### 2.4. Statistical Methods

We compared and analyzed real-time PCR results using a Kruskal–Wallis test among groups and Wilcoxon two group test to calculate pairwise comparisons between each group and proximal big sucker result, *p*-values < 0.05 were considered statistically significant.

### 2.5. Sucker Whole-Mount In Situ Hybridization 

To generate Ov-Rhodopsin_kinase (Ov-GRK1) Digoxigenin (DIG)-labeled single-stranded RNA probes, we performed PCR standard method using a specific primer set (Table 1). The PCR fragments were used as templates for in vitro transcription reaction using the T7 RNA polymerase promoter sequence corresponding to forward and reverse primers for the sense and antisense probes, respectively. PCR cDNA fragments were isolated by 1.2% agarose gel and were used as templates for in vitro transcription reactions. An RNA transcription reaction was performed using the DIG-RNA Labelling Kit (SP6/T7) (Roche Applied Sciences, Laval, QC, Canada) following the manufacturer’s instructions. Final probes were cleaned up using RNeasy MinElute Cleanup Kit (Qiagen, GmbH Valencia, CA, USA), and one microliter was visualized on a 1.5% agarose gel to estimate concentration.

For whole mount in situ hybridization, fixed suckers were rehydrated by descending methanol series in 75%, 50%, and 25% MeOH in PBST for 15 min each at RT. Completed rehydration was performed twice in 100% PBST for 10 min each with gentle rocking. Tissues were incubated and digested in the detergent mix (20 µg/mL in PBST Proteinase-K) at 37 °C for 20 min, post-fixed in 4% PFA for 20 min at room temperature, then washed three times in PBST for 5 min each. Tissues were pre-hybridized for 2 h and hybridized overnight at 62 °C in hybridization solution (50% formamide, 5X saline-sodium citrate (SSC), 1X Denhardt’s solution, 500 mg/mL yeast tRNA and 500 mg/mL salmon sperm DNA). After hybridization, the tissues were incubated with 20 µg/mL RNAs A (Invitrogen 12091021) for 15 min at 37 °C, then subjected to a series of post-hybridization washes in decreasing concentrations of SSC with 0.1% Tween 20. Tissues were blocked in 1X blocking solution (Roche Applied Science 11096176001) in PBST for 1 h at room temperature under gentle rocking, followed by incubation in 1:2500 Anti-Digoxigenin-AP antibody (Roche) in blocking solution overnight at 4 °C on a rocker. Tissues washed in PBST five times for 25 min each, equilibrated in alkaline phosphatase buffer (AP) (100 mM NaCl, 50 mM MgCl_2_, 100 mM Tris, pH 9.5, 0.1% Tween-20) at room temperature. The color reaction was performed in NBT/BCIP stain solution (Roche) in AP buffer under the light-resistant environment until the colors reached a satisfactory intensity. Control specimens were left in staining solution for the same time interval as those incubated with anti-sense probes. In order to test for nonspecific labeling, negative control experiments were performed for each condition using only hybridization buffer without probe.

After the coloration reaction, all tissues were passed through ascending concentrations of ethanol in PBS to remove background and darken the specific signal, re-hydrated in PBS.

Whole-mount sucker tissues with DIG-labeled probes were observed colorimetrically under a Carl Zeiss Stemi 305 stereomicroscope with Axiocam Erc 5 s.

### 2.6. Molecular Phylogenetic Analysis

To construct the evolutionary relationships of the Rhodopsin kinase receptor we aligned the sequence of *O. vulgaris* kinase (Ov-GRK1, XM_029795790.1) with those of other amino sequences of vertebrate Rhodopsin kinase (RK) and Bilaterian GPCR Kinases, together with 6 previously identified cephalopod RK sequences, including *Enteroctopus dofleini, Loligo forbesii, Doryteuthis pealeii, Euprymna scolopes* and *O. bimaculoides* (Supplementary Table S1).

Protein sequences were aligned with the MUSCLE algorithm [36], included in the software package MEGAX [37] with default parameters. ProtTest v3.4.2 was used to establish the best evolutionary model [38]. Bayesian tree was constructed using MrBayes v3.2.7 [39] and Bayesian inference phylogenies were run for 1,000,000 generations. Markov chain Monte Carlo (MCMC) was used to approximate the posterior probability of the Bayesian trees. Bayesian analyses included four independent MCMC runs, each using four parallel chains composed of three heated and one cold chain. Ten per cent of initial trees were discarded as burn-in. Phylogenetic trees were rendered using FigTree (http://tree.bio.ed.ac.uk/software/figtree [40], accessed on 30 January 2021 ). 

### 2.7. Prediction of 3D Structure Model

To estimate sequence similarities of OV-GRK1 protein, the GRK1 protein sequence of *O. bimaculoides* (XP_014774259) was aligned with rhodopsin kinase sequences of the light organ (ACB05677) and the eye (ACB05676) of *E. scolopes*. The alignment was performed using CLUSTALW [41] and colored according to the CLUSTALX scheme using JALVIEW [42].

Homology modeling of the Ov-GRK1 protein structure was performed using the SWISS-MODEL Web server (http://swissmodel.expasy.org, accessed on 30 January 2021) (Supplementary Figures S1 and S2 Supplementary Material online). The three-dimensional (3D) structure of Ov-GRK1 protein was built based on the target-template alignment using ProMod3 [43]. The human G-protein coupled receptor kinase 2 [44] (PDBe ID: 6c2y) was selected as the template (sequence similarity: 66.3% and The QMEAN Z-score: 1.26). The target sequence was searched with BLAST and HHBlits against the primary amino acid sequence contained in the SMTL at the SWISS-MODEL template library [45,46,47]. The global and per-residue model quality was assessed using the QMEAN scoring function [48].

To visualize the predicted model, the graphical representations of the protein structures for the Ov-GRK1 structure was created using PyMOL (version 1.3) (DeLanoScientific, San Carlos, CA). Additionally, the physiochemical characteristics such as half-life, number of amino acids, theoretical *pI*, and extinction coefficient for the Ov-GRK1 protein were predicted by the ProtParam tool (https://web.expasy.org/protparam, accessed on 30 January 2021).

## 3. Results

### 3.1. Sequencing and Expression Analysis of Ov-GRK1 Gene

**Figure 2:**
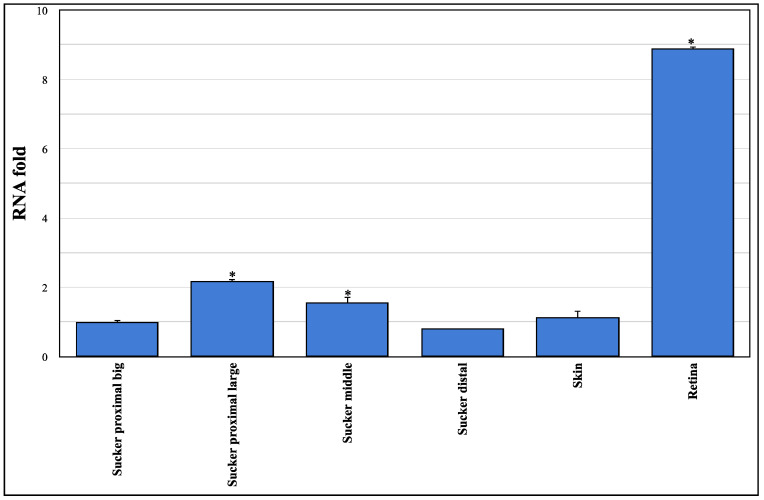
Gene expression analysis for mRNA of Rhodopsin kinase receptor gene (Ov-GRK1) of different tissues. Relative mRNA expression levels were measured using real-time analysis and calculated by the 2^(−ΔΔC(T))^ method. Each sample (*n* = 3) was tested and run in duplicate. The heart tissue was used as a negative control. The ubiquitin gene was amplified as an internal control. No-template controls were included. Relative mRNA fold change in gene expression was compared to the proximal big sucker (set y = 1). * asterisk indicates that the difference vs. sucker proximal big is statistically significant (Wilcoxon-test, *p* < 0.05). The Kruskal–Wallis test showed that there was a statistically significance between groups (*p* < 0.05). Error bars represent the SEM.

Here, we present molecular evidence of the *Ov-GRK1* gene expression in the epithelium rim of different types of *O. vulgaris* suckers (Figure 2).

RT-PCR amplification revealed that the *Ov-GRK1* gene is expressed in the suckers and in the skin and retina of *O. vulgaris*. There is a significant difference in the gene expression among different types of suckers: up-regulated in distal big and middle suckers than distal large ones; meanwhile, there are no significant differences in the gene expression among skin, suckers distal big and proximal ones. *Ov-GRK1*gene expression in the retina tissue results about 9-fold larger than in distal big suckers. The heart tissue did not show any expression and was used as a negative control.

### 3.2. Localization of Ov-GRK1 Transcript in the Sucker of O. vulgaris

**Figure 3:**
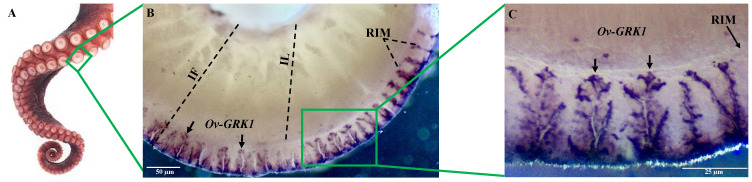
Whole-mount in situ hybridization of the Ov-GRK1 gene in the rim of an *O. vulgaris* middle sucker. (**A**) Octopus arm with array of suckers; (**B**) the receptor expression is present in the epithelium of the rim (arrows); (**C**) higher magnification of the portion of the epithelium rim inside the green rectangle showing Ov-GRK1 expression. Sucker rim epithelium (RIM), sucker infundibulum (IF), infundibulum lumen (IL). Arrows indicate positive signals.

In order to localize Ov-GRK1 transcript in the sucker of *O. vulgaris*, we performed the whole-mount in situ hybridization with probes constructed on the *Ov-GRK1* gene (Figure 3). *Ov-GRK1* gene expression was exclusively located around the outer border of the epithelium rim (RIM), but no expression was detected in the epithelium lining of the sucker infundibulum (IF) (Figure 3B). This receptor expression is widely and regularly distributed around the epithelium of the rim (Figure 3B,C). We observed an unusual, branched shape (Figure 3C), with high expression in the external portion of the rim. From the outer part of the rim it spreads in numerous finger-like and laminar projections into the inner part of the rim (Figure 3C).

### 3.3. Molecular Phylogenetic Construction

**Figure 4:**
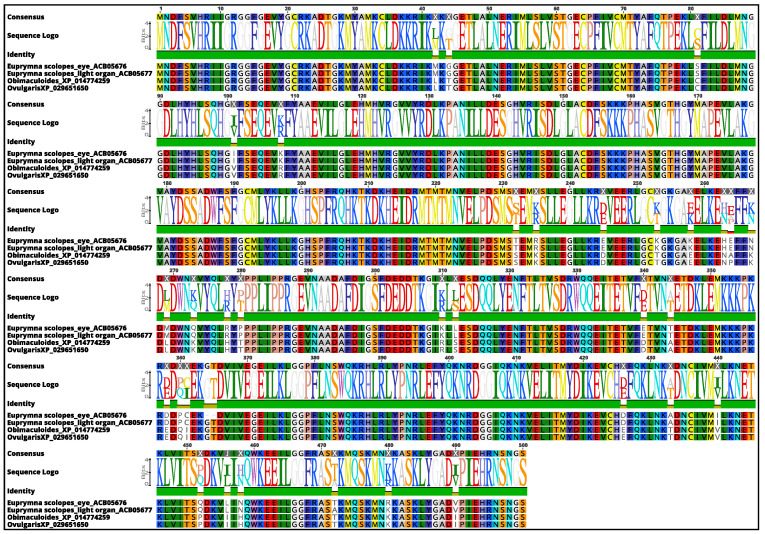
GRK1 amino acid sequences alignment. Alignment of Ov-GRK1 amino acid sequences to an *O. bimaculoides* rhodopsin kinase amino acid sequence from GenBank (accession number XP014774259) and to those of *E. scolopes* rhodopsin kinase extracted from the eyes (ACB05676) and light organ (ACB05677). White line boxes highlight potential amino acid differences among samples. Complete open reading frames are shown.

The sequence analysis showed that rhodopsin kinases from *O. vulgaris* and *O. bimaculoides* were almost identical (99.80% sequence identity). Moreover, *E. scolopes* rhodopsin kinase, identified in the eyes (ACB05676.1) and light organ (ACB05677.1), also showed a high similarity with the rhodopsin kinase of *O. vulgaris* (92.33% and 92.04% of sequence identity, respectively), revealing a high similarity in genes among species (Figure 4).

**Figure 5:**
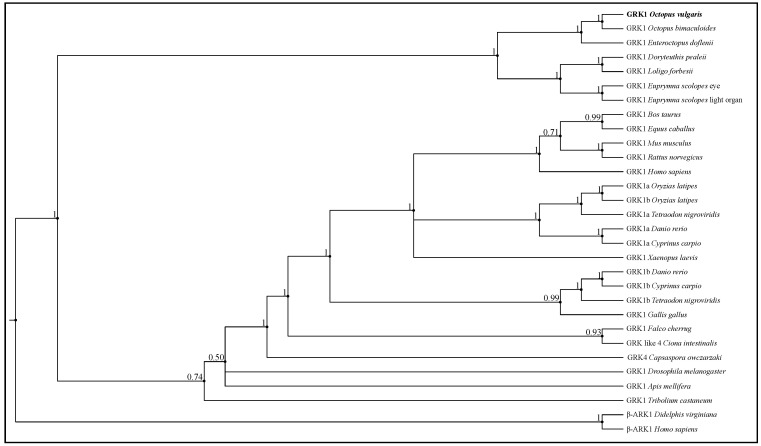
Bayesian tree of GRK protein sequences. Bayesian phylogenetic tree performed with LG + G model, constructed with protein sequences from Ov-GRK1 and Rhodopsin kinase receptors of 18 species including other cephalopods, invertebrates and vertebrates. All protein sequences were obtained from GenBank and the accession numbers are presented in Table S1. Bayesian posterior probabilities are represented over nodes; turquoise box highlights the cephalopod clade and the Ov-GRK1 protein sequence is in bold.

The phylogenetic tree shows that Ov-GRK1 is closely related to other GRK1 identified in other cephalopods, including *O. bimaculoides* and *E. dofleini* (Figure 5).

In particular, the Ov-GRK1 gene in cephalopods is clearly evolutionary distinct from the ecdysozoans and vertebrates GRK1 family.

### 3.4. Prediction of the 3D Structure Model for Ov-GRK1

**Figure 6:**
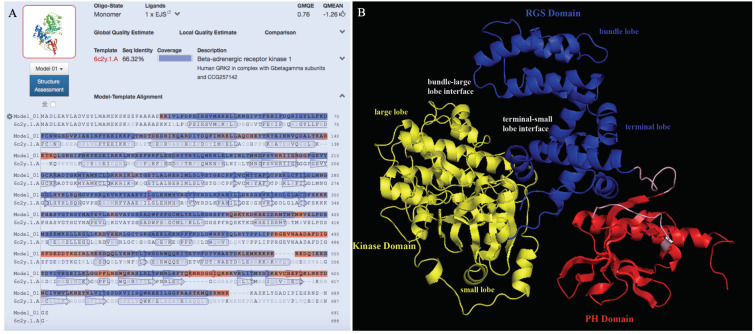
Homology modeling of the Ov-GRK1. (**A**) Alignment with a potential Model-Template (6c2y). (**B**) 3D structural view for the prediction of ligand binding sites in Ov-GRK1 generated by 3DLigandSite based on the PDB.

We created a novel 3D model structure for Ov-GRK1 protein (Figure 6). None of the proteins reported in the previous section of the article bearing a sequence identity greater than 90% were available in the PDB database. Therefore, the model was constructed based on the crystal structure of a G-protein coupled receptor kinase 2 (GRK2) (PDBe ID: 6c2y), which showed the maximum sequence identity (66.3%) to the query protein Ov-GRK1 (Figure 6A). GRK2 in humans is an enzyme encoded as the adrenergic-beta-receptor kinase 1 (ADRBK1) gene and it was initially called Beta-adrenergic receptor kinase (βARK or βARK1). Our results showed that the Beta-adrenergic receptor kinase (β-ARK1; modern name GRK2) was the best template obtained with BLAST and HHBlits (lightning-fast iterative protein sequence searching by HMM-HMM alignment) against the primary amino acid sequence contained in the SWISS-MODEL template library (SMTL). Ov-GRK1 has been modeled with a high accuracy (coverage: 0.98; the global model quality estimation (GMQE); score: 0.77) using a single highest-scoring template.

The obtained model is characterized by the presence of three main domains: the regulator of G-protein signaling (RGS) domain (Figure 6B, colored in blue), which is located at the N terminal side and is homologous to that found in the regulator of G protein signaling family of proteins [49]. This comprises two subdomains called the bundle lobe and the terminal lobe. The kinase domain (Figure 6B, colored in yellow) comprises two subdomains: the small and large lobe and the pleckestrin domain (PH) (Figure 6B, colored in red), which is responsible for the interaction with G protein β γ subunits and plasma membranes upon phosphorylation of the substrate receptors. There are two distinct interfaces between the RGS and kinase domains; the larger contact interface is between the terminal lobe of RGS and the small lobe of the kinase domain, with a sequence identity and conservation between β-ARK1 and Ov-GRK1 which is extremely high. The smaller interface is between the bundle lobe of RGS and the large lobe of the kinase and this contact area is highly conserved. The terminal lobe of RGS also forms an extensive contact interface with the PH domain, which is also highly conserved. The C terminal domain of the protein, located in the domain PH is not structured as expected, because it is probably ordered upon interaction with G β γ subunits. The kinase domain is highly conserved while the RGS and PH domains present the main sequence differences. This finding agrees with previous work which found that the RGS and PH domains of β-ARK1 move as a single domain with respect to the kinase domain between the active and inactive structures [50]. The highest sequence differences, characterized by deletions and insertions of amino acids, are located in the PH domain in a region of high conformational variability (Figure 6B, colored in red). These differences are not contiguous in the sequence but constitute a surface, which is likely to be characteristic too.

The predicted molecular weight of the Ov-GRK1 was estimated as 79.6 kDa by the ProtParam tool (https://web.expasy.org/protparam, accessed on 30 January 2021 ), which is similar to that of β-ARK (80 kDa) [51] (File S2). Additionally, the physicochemical characteristics such as half-life, number of amino acids, theoretical *pI* (isoelectric point), and extinction coefficient for Ov-GRK1 protein were predicted (File S2).

## 4. Discussion

We found Ov-GRK1 expression in the retina, skin and suckers of *Octopus vulgaris.* By now we know that sensory receptors originally identified in specific sensory organs are often found in other areas in the body. In many animals, the ability to detect and use light for different biological purposes is mediated by visual pigment molecules that act as light sensors for visual and non-visual functions [52,53]. In cephalopods, a variety of photoreceptor molecules have been found outside their classical sensory organs, where they respond to different stimuli, initiating signaling cascades in these extraocular systems. There are several well-studied extraocular photoreceptors including rhodopsin in the light organ of several cephalopods, the parolfactory vesicles of squids, and the opsins (rhodopsin, retinochrome, Gq-coupled opsin) in cephalopod skin that are believed to intrinsically contribute to light detection and to dermal patterning [9,11,14,16,17,54,55,56,57]. These suggest the presence of extraocular “vision” in these animals.

In octopuses, the eight highly flexible arms are the main interaction points between animals and the outside world. These arms have been shown to be both centrally controlled when needed, and extremely independent with extensive reflex loops coordinating local motion. Most sensory cells are found in the epithelium rim of the sucker, and Graziadei and Gagne [24,58] described four morphological types of primary receptors and postulated on their function. For one of these, termed “an unusual receptor” (designated as Cell Type 3 in [24]), they suggested “for the microvillus formation could be construed as having some relation to the rhabdomeres of light sensitive cells” [59]. Our whole mount in situ hybridization of Ov-GRK1 in the sucker rim epithelium revealed a structure closely resembling the type 3 receptors described by Graziadei and Gagne [24,58].

We showed a significant differential expression of the Ov-GRK1 mRNA in selected suckers in different locations along the arm (Figure 2). Interestingly, there is no significant difference in the gene expression of the distal suckers at the tip of the arm, and in the octopus skin. Not surprisingly, the Ov-GRK1 expression results are highly up-regulated in the retina, approximately nine-fold more than in the highest expressing suckers, the proximal large sucker.

Behavioral research describing various types of arm movements in the octopus, such as the motor primitive reaching and fetching, maze experiments and peripheral search movements, all indicate that different areas along the arm tend to be used differently. Thus, our results regarding the differentiated expression of Ov-GRK1 related to the position along the arm might correlate to these various functions.

Hunting behavior in *Octopus vulgaris* often involves inserting arms into crevices, out of the field of vision. This movement probably involves following stimuli encountered by the arm, which adhere to and release from surfaces and explore crevices to “scare out” food. Arm insertion into crevices often involved creating a bend, with the suckers facing out, at the approximate location in which we found higher expression of Ov-GRK1 in the suckers, this might indicate functional extra-ocular light sensing perception in addition to touch and taste senses [18]. This capability could affect the predatory performance of octopuses, allowing them to track down a hidden prey, detecting their presence in dark burrows due to their bioluminescence [60,61]. The diffuse light sensing ability could have a significant role in their ability to change color to camouflage through background matching, guiding the highly specialized chromatic elements in their skin to display complex and rapidly changing color patterns at the peripheral level, without using the central nervous system, speeding up the response to environmental changes, as observed for other species [62,63,64,65,66]. Interestingly, the enlarged suckers found in the same area of the arm did not express a higher level of Ov-GRK1, these suckers, only found in males, probably have other specific roles. These differences stimulate further research to explore the light sensing abilities of suckers along the arms.

The recent availability of genomic sequencing data for the four octopus species, *O. bimaculoides* [29], *O. vulgaris* [67], *O. minor* [68], and *O. sinensis* [69], allowed us to identify the rhodopsin kinase (GRK1) in *O. vulgaris*. This molecule is a member of G protein-coupled receptors that detect light. It is found primarily in mammalian retinal rod cells, where it phosphorylates light-activated rhodopsin, and is officially named G protein-coupled receptor kinase 1, or GRK1.

Previous studies of the transcriptome of the light organ in *E. scolopes* have shown that the expression of several genes encoding visual transduction proteins includes the same isoform of opsin that occurs in the retina [11]. In our study, the percentage identity and similarity among the amino acid sequences of rhodopsin kinase in the epithelium sucker rim of *O. vulgaris*, the light organ, and retina of *E. scolopes* revealed over 92% sequence identity.

The phylogenetic tree obtained using the Bayesian method (Figure 5) clearly shows that Ov-GRK1 appears to be, along with another cephalopod rhodopsin kinase branch, in the same clade, and not surprisingly results in the nearest neighbor to rhodopsin kinase receptor described in *O. bimaculoides*.

Additionally, in an effort to elucidate the molecular and enzymatic properties of Ov-GRK1 in the octopus sucker, we predicted the in silico 3D structure of Ov-GRK1 protein, for the first time, based on the homology modeling technique. The availability of an Ov-GRK1 protein 3D structure is one of the keys for a deeper understanding of the biological processes it underlies at a molecular and structural level. The maximum sequence identity with a protein present in the PDB database was obtained between Ov-GRK1 and GRK2 (66.3%). GRK2 is an enzyme in humans which is encoded by the ADRBK1 gene and it was initially called Beta-adrenergic receptor kinase (βARK or βARK1) (Figure 6A). It has been demonstrated that many G protein-coupled receptors, such as βARK and rhodopsin kinase (RK), are phosphorylated in a light- or agonist-dependent manner, by a member of the specific protein kinase family called G protein-coupled receptor kinases (GRKs) [70,71]. Desensitisation of these receptors is thought to be related to stimulus-dependent phosphorylation of the area [71,72].

In our study, the 3D model we created is characterized by the presence of three main domains: the RGS domain (Figure 6B, colored in blue), which comprises two subdomains referred to as the bundle lobe and the terminal lobe; the protein kinase domain (Figure 6B, colored in yellow), which comprises two subdomains called the small and large lobe; and the PH domain (Figure 6B, colored in red), which is responsible of the interaction with G protein G β γ -subunits and plasma membranes upon phosphorylation of substrate receptors. Our results are similar to those obtained in a previous study in retina of *O. dofleini* in which it is reported that octopus rhodopsin kinase is markedly enhanced by GTP [73], suggesting that it could be activated by G β γ -subunits of a photoreceptor G protein.

Rhodopsin kinases 3D structure is well known in mammalians. Rhodopsin kinase (GRK1) and β-adrenergic receptor kinases (GRK2/3 or β-ARK1/2) are both responsible for phosphorylating the activated forms of rhodopsin and β-adrenergic receptors. The sequence similarity between the two kinases is not very high, with the main differences located in the C-terminal region. In particular, GRK1 lacks the pleckestrin homology domain (PH) at the C-terminus (approximately 120 residues). While β-ARK interacts with and is activated by Gβγ, GRK1 is not affected by Gβγ and instead contains at the C-terminus the motif termed CAAX. Thus, β-ARK1 and GRK1 represent different subgroups in the GRK family in terms of structure and regulatory mechanisms. It is interesting to note that the PH domain-like sequence in the C-terminal region, which is the domain involved in the interaction with the G protein βγ-subunit, is present in β-ARK but not in GRK1 [71,72]; and the PH domain is also present in Ov-GRK1. The sequence alignment between the PH domain of Ov-GRK1 and β-ARK1 clearly shows that Ov-GRK1 contains some extra residues DGGIQKNKV, which are missing in the other sequence and may be related to some features specific to *O. vulgaris*.

Since light depolarizes invertebrate photoreceptor cells, whereas it hyperpolarizes vertebrate rod and cone photoreceptor cells, the underlying phototransduction machinery, including that for desensitization in invertebrate photoreceptor cells, could be quite distinct from that of vertebrate photoreceptors [74]. These findings are partially similar to the previous studies that have reported that GRK1 regulates mammalian cone phototransduction during photoreceptor light adaptation [75,76]. Furthermore, the predicted molecular weight of the Ov-GRK1 is estimated to be 79.6 kD, which is also consistent with the molecular mass predicted from the sequence of rhodopsin kinase retinal photoreceptor in *Octopus dofleini* (80 kDa) [77].

## 5. Conclusions

Using a combination of techniques, we show the complete characterization of Ov-GRK1 in several tissues of *O. vulgaris*. The sequence, its phylogenetic relation, the differential expression pattern and localization, together with the 3D structure, suggest that the Ov-GRK1 is an extra-ocular photoreceptor type, allowing for diffused light-sensing capabilities in specific areas of the octopus arms. These findings expand our knowledge of extra-ocular sensing in the octopus, beyond generalized skin and light organ reaction to suckers in specific arm areas, suggesting a functional use. Future experiments should be developed using physiological and behavioral techniques to strengthen the hypothesis of the extra-ocular visual perception with arm. Our findings show that octopus suckers have the molecular machinery, and the physiological potential to respond to light cues, adding a new sensory dimension to their previously shown multimodal sensory capabilities.

## Figures and Tables

**Table 1:** Primers used in this study.

**Primer Pairs Used in RT-PCR**	**Primer Sequences (5′→3′)**
Ov-GRK1 F	CCGCCTCTCATTCCTCCAAG
Ov-GRK1 R	AGATCTCTCCTTCCACAATCACA
Ubiquitin_ F	TCAAAACCGCCAACTTAACC
Ubiquitin_ R	CCTTCATTTGGTCCTTCGTC
**For WM-ISH probe**	**Primer sequences (5′→3′)**
Ov-GRK1 F	CCGCCTCTCATTCCTCCAAG
Ov-GRK1 R + T7	*TAATACGACTCACTATAGGGGAGA* AGATCTCTCCTTCCACAATCAC

## Data Availability

Data are contained within the article or Supplementary Materials.

## Supplementary Materials

The following are available online at https://www.mdpi.com/article/10.3390/biology10090936/s1, S1—Sequence Alignments and Homology model of Ov-GRK1 including Supplementary Table. S2—Physicochemical characteristics of Ov-GRK1.
