# Supplementary material for: Identification and Characterization of a Rhodopsin Kinase Gene in the Suckers of Octopus vulgaris: Looking around Using Arms?
Source: Biology (Basel). 2021 Sep 19;10(9):936. doi: 10.3390/biology10090936 (PMC8465341; doi:10.3390/biology10090936)
Supplement: Supplementary file 1 [file biology-10-00936-s001.zip › Supplementary material/S1.pdf]

# A0A6P7TP13 (A0A6P7TP13_OCTVU) *Octopus vulgaris (Common octopus)*

## G protein-coupled receptor kinase ☆ UniProtKB InterPro Interactive Modelling

691 aa; Sequence (Fasta)

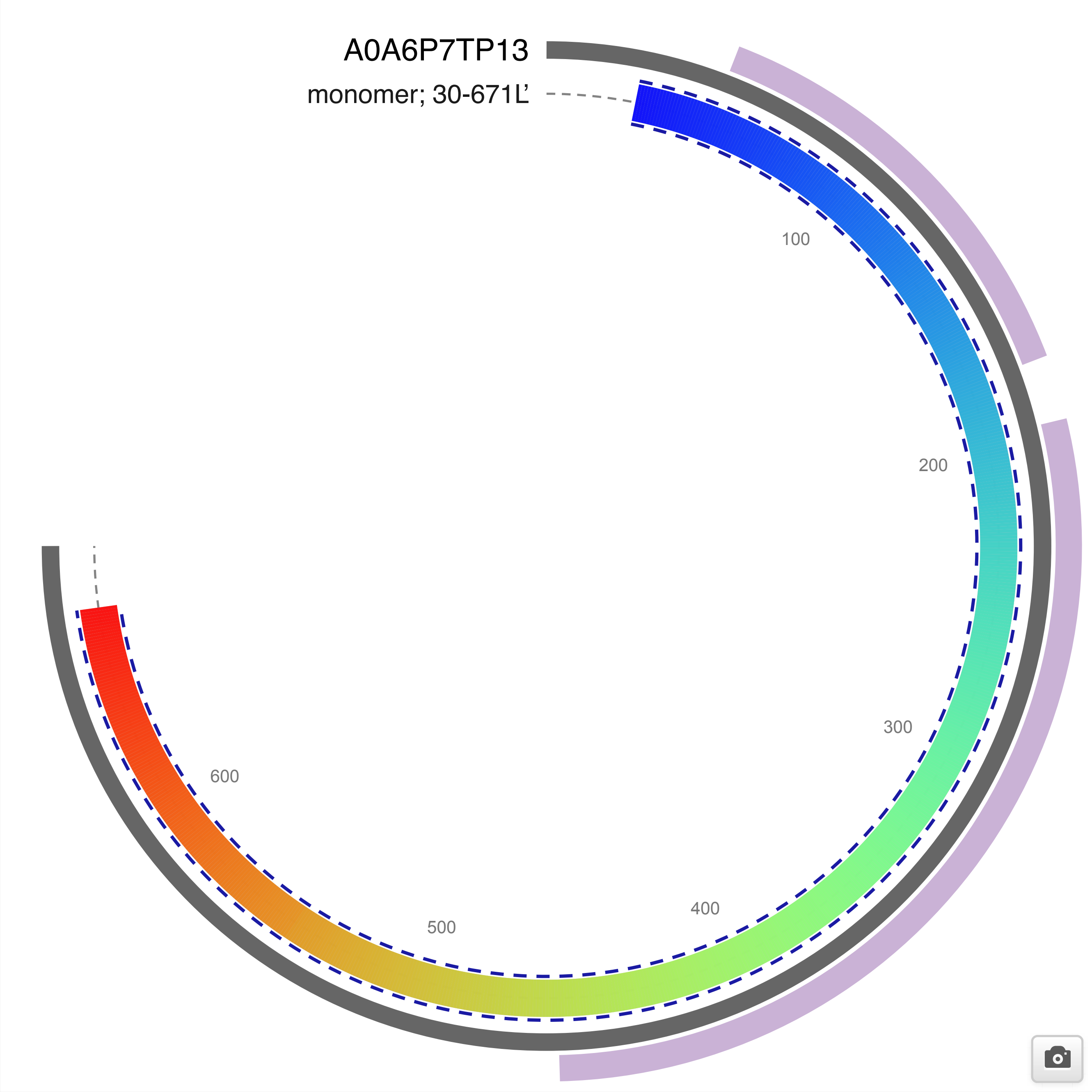


### Homology Model

Template: **6c2y.1.A** *"Human GRK2 in complex with Gbetagamma subunits and CCG257142"* *P25098*

SMTL Version: 2021-03-03

Seq Identity: 66.32%

Seq Similarity: 0.50

1 x (4R,5R,6S)-4-[4-fluoro-3-({[3-(methoxymethyl)-1,2,4-oxadiazol-5-yl]methyl}carbamoyl)phenyl]-N-(2H-indazol-5-yl)-6-methyl-2-oxohexahydropyrimidine-5-carboxamide

Coordinates:

### Model Quality Estimate

| | |
|---|---|
| QMEAN | -1.26 |
| Cβ | -1.30 |
| All Atom | -0.18 |
| solvation | 0.79 |
| torsion | -1.28 |

### Sequence Features

InterPro + Add

Cartoon

## Sequence Alignments

### *Model based on 6c2y.1.A Beta-adrenergic receptor kinase 1*

```
    MODEL KKIVLPDPSIRSVMHKHLLKNGIVTFEHIFDQRIGYLLFKDFCNNGSDVPIAEINFYEEIKKFQTMDTDEDRIKQARDIY 109
 6c2y.1.A KKILLPEPSIRSVMQKYLEDRGEVTFEKIFSQKLGYLLFRDFCLNHLEEARPLVEFYEEIKKYEKLETEEERVARSREIF 109

    MODEL DQFIMRELLAQCHEYTKTAINNVQDALTKARKTKQLGNHIFNKYKDEIRKKLNKEEFNKFLESDRYTRYLQWKNLELNIN 189
 6c2y.1.A DSYIMKELLACSHPFSKSATEHVQGHLGK----KQVPPDLFQPYIEEICQNLRGDVFQKFIESDKFTRFCQWKNVELNIH 185

    MODEL LTMNDFSVHRIIGRGGFGEVYGCRKADTGKMYAMKCLDKKRIKLKTGETLALNERIMLSLVSTGECPFIVCMTYAFQTPE 269
 6c2y.1.A LTMNDFSVHRIIGRGGFGEVYGCRKADTGKMYAMKCLDKKRIKMKQGETLALNERIMLSLVSTGDCPFIVCMSYAFHTPD 265

    MODEL KLCFILDLMNGGDLHYHLSQHGVFSEQEVRFYAAEVILGLEHMHVRGVVYRDLKPANILLDESGHVRISDLGLACDFSKK 349
 6c2y.1.A KLSFILDLMNGGDLHYHLSQHGVFSEADMRFYAAEIILGLEHMHNRFVVYRDLKPANILLDEHGHVRISDLGLACDFSKK 345

    MODEL KPHASVGTHGYMAPEVLAKGVAYDSSADWFSFGCMLYKLLKGHSPFRQHKTKDKHEIDRMTMTMNVELPDSMSSEMKSLL 429
 6c2y.1.A KPHASVGTHGYMAPEVLQKGVAYDSSADWFSLGCMLFKLLRGHSPFRQHKTKDKHEIDRMTLTMAVELPDSFSPELRSLL 425

    MODEL EGLLKRDVEERLGCTGKGAEELKENPFFKDLDWNKVYQLHYTPPLIPPRGEVNAADAFDIGSFDEDDTKGIRLSESDQQL 509
 6c2y.1.A EGLLQRDVNRRLGCLGRGAQEVKESPFFRSLDWQMVFLQKYPPPLIPPRGEVNAADAFDIGSFDEEDTKGIKLLDSDQEL 505
```

## Homology models

| Oligo-state | QMEAN | Template | Range | Seq id (%) | Ligands |
|---|---|---|---|---|---|
| monomer Assess | -1.26 | 6c2y.1.A | | 66.32 | 1×EJS; |
