# Supplementary material for: Identification and Characterization of a Rhodopsin Kinase Gene in the Suckers of Octopus vulgaris: Looking around Using Arms?
Source: Biology (Basel). 2021 Sep 19;10(9):936. doi: 10.3390/biology10090936 (PMC8465341; doi:10.3390/biology10090936)
Supplement: Supplementary file 1 [file biology-10-00936-s001.zip › Supplementary material/S2- ExPASy.pdf]

ProtParam

A0A6P7TP13_OCTVU (A0A6P7TP13)

G protein-coupled receptor kinase (EC 2.7.11.-)
Octopus vulgaris (Common octopus)

The computation has been carried out on the complete sequence (**691** amino acids).

Warning: All computation results shown below do **not** take into account any annotated post-translational modification.
References and documentation are available.

Number of amino acids: 691

Molecular weight: 79613.46

Theoretical pI: 7.58

Amino acid composition: CSV format

Ala (A)	34	4.9%
Arg (R)	33	4.8%
Asn (N)	33	4.8%
Asp (D)	47	6.8%
Cys (C)	12	1.7%
Gln (Q)	24	3.5%
Glu (E)	54	7.8%
Gly (G)	41	5.9%
His (H)	22	3.2%
Ile (I)	42	6.1%
Leu (L)	66	9.6%
Lys (K)	69	10.0%
Met (M)	25	3.6%
Phe (F)	31	4.5%
Pro (P)	21	3.0%
Ser (S)	37	5.4%
Thr (T)	34	4.9%
Trp (W)	6	0.9%
Tyr (Y)	24	3.5%
Val (V)	36	5.2%
Pyl (O)	0	0.0%
Sec (U)	0	0.0%
(B)	0	0.0%
(Z)	0	0.0%
(X)	0	0.0%

Total number of negatively charged residues (Asp + Glu): 101
Total number of positively charged residues (Arg + Lys): 102

Atomic composition:

Carbon	C	3540
Hydrogen	H	5598
Nitrogen	N	966
Oxygen	O	1046
Sulfur	S	37

Formula: $C_{3540}H_{5598}N_{966}O_{1046}S_{37}$
Total number of atoms: 11187

Extinction coefficients:

Extinction coefficients are in units of $M^{-1} cm^{-1}$, at 280 nm measured in water.

Ext. coefficient 69510
Abs 0.1% (=1 g/l) 0.873, assuming all pairs of Cys residues form cystines

Ext. coefficient 68760
Abs 0.1% (=1 g/l) 0.864, assuming all Cys residues are reduced

Estimated half-life:

The N-terminal of the sequence considered is M (Met).

The estimated half-life is: 30 hours (mammalian reticulocytes, in vitro).
>20 hours (yeast, in vivo).
>10 hours (Escherichia coli, in vivo).

Instability index:

The instability index (II) is computed to be 42.42
This classifies the protein as unstable.

Aliphatic index: 80.98

Grand average of hydropathicity (GRAVY): -0.527
