# Supplementary material for: Identification and Characterization of a Rhodopsin Kinase Gene in the Suckers of Octopus vulgaris: Looking around Using Arms?
Source: Biology (Basel). 2021 Sep 19;10(9):936. doi: 10.3390/biology10090936 (PMC8465341; doi:10.3390/biology10090936)
Supplement: Supplementary file 1 [file biology-10-00936-s001.zip › Supplementary material/Supplementary Table S1.pdf]

Organism (GI No.)	Gene Name	Full name	GenBank Accession No.
Ciona intestinalis	GRK4	similar to G protein-coupled receptor kinase 4	XP_002122095
Apis mellifera	G protein-coupled receptor kinase 1	similar to G protein-coupled receptor kinase 1 isoform 1	XP_396647
Bos taurus	GRK1	rhodopsin kinase GRK1	NM_174173
Capsaspora owczarzaki ATCC 30864	GRK4	G protein-coupled receptor kinase 4	EFW41776
Cyprinus carpio	rhodopsin kinase GRK1a	G protein-coupled receptor kinase 1a	BAB32497
Cyprinus carpio	rhodopsin kinase GRK1b	G protein-coupled receptor kinase 1b	BAE66638
Danio rerio	rhodopsin kinase GRK1a	rhodopsin kinase GRK1a	NP_001029353
Danio rerio	rhodopsin kinase GRK1b	rhodopsin kinase GRK1b	NP_001017711
Didelphis virginiana	ARBK1_DIDVI	Beta-adrenergic receptor kinase 1	O97627
Doryteuthis pealeii	rhodopsin kinase	rhodopsin kinase	AAF08967
Drosophila melanogaster	GRK1	G protein-coupled receptor kinase 1, isoform A	NP_001036438
Enteroctopus dofleini	rhodopsin kinase	rhodopsin kinase	BAA75507
Equus caballus	rhodopsin kinase	rhodopsin kinase	XP_001504141
Euprymna scolopes	light organ rhodopsin kinase	light organ rhodopsin kinase mRNA	ACB05677
Euprymna scolopes	eye rhodopsin kinase	eye rhodopsin kinase mRNA	ACB05676
Falco cherrug	rhodopsin kinase	rhodopsin kinase	XP_005443720
Gallus gallus	rhodopsin kinase GRK1	rhodopsin kinase GRK1	NP_990026
Homo sapiens	GRK1_HUMAN	G protein-coupled receptor kinase 1	Q15835
Homo sapiens	ARBK1_HUMAN	Beta-adrenergic receptor kinase 1	P25098
Loligo forbesii	rhodopsin kinase	rhodopsin kinase	AAR19398
Mus musculus	GRK1	rhodopsin kinase GRK1	NP_036011
Octopus bimaculoides	G protein-coupled receptor kinase 1	G protein-coupled receptor kinase 1-like	XP_014774259
Octopus vulgaris	G protein-coupled receptor kinase 1	G protein-coupled receptor kinase 1-like	XP_029651650
Oryzias latipes	GRK1a	OlGRK-R [Oryzias latipes]	BAA25671
Oryzias latipes	GRK1b	OlGRK-R (Rhodopsin kinase GRK1 [Oryzias latipes])	NP_001188419
Rattus norvegicus	GRK1	rhodopsin kinase GRK1 [Rattus norvegicus]	NP_112358
Tetraodon nigroviridis	GRK1a	G protein-coupled receptor kinase 1-like	47230559
Tetraodon nigroviridis	GRK1b	G protein-coupled receptor kinase 1-like	47222525
Tribolium castaneum	G protein-coupled receptor kinase 1	G protein-coupled receptor kinase 1 [Tribolium castaneum]	XP_966480
Xenopus laevis	GRK1	LOC733223 protein, partial [Xenopus laevis]	AAH94133
